# Secondary headaches attributed to arterial hypertension

**Published:** 2013

**Authors:** Farhad Assarzadegan, Mostafa Asadollahi, Omid Hesami, Omid Aryani, Behnam Mansouri, Nahid Beladi moghadam

**Affiliations:** 1 Assistant Professor, Department of Neurology, Imam Hossein Hospital, Shahid Beheshti University of Medical Sciences, Tehran, Iran; 2 Neurologist, Special Medical Center, Tehran, Iran

**Keywords:** Hypertension, Headache, Hypertensive Encephalopathy, Eclampsia, Pheochromocytoma

## Abstract

Mild (140 to 159/90 to 99 mmHg) or moderate (160 to 179/100 to 109 mmHg) chronic arterial hypertension does not appear to cause headache. Whether moderate hypertension predisposes patients to headache at all remains controversial, but there is little evidence that it does. Ambulatory blood pressure monitoring in patients with mild and moderate hypertension has shown no convincing relationship between blood pressure fluctuations over a 24-hour period and presence or absence of headache. However, headaches are associated to various disorders that lead to abrupt, severe, and paroxysmal elevations in blood pressure. In this paper, the secondary headaches attributed to acute crises of hypertension and the criteria for diagnosing each of them have been reviewed. These are headaches attributed to pheochromocytoma, hypertensive crisis without encephalopathy, hypertensive encephalopathy, pre-eclampsia, eclampsia, and acute pressure response to exogenous agents.

## Introduction

Among the vast variety of secondary headaches, a considerable group is related to high blood pressure. Commonly, an abrupt elevation of arterial blood pressure is responsible for headache rather than the absolute value. HTN attributed headaches are discussed under 5 major categories and for each one definite criteria have been validated [International Headache Classification (IHCD-2)]^1^:

1. Pheochromocytoma (benign or malignant)
2. HTN crisis without encephalopathy
3. Hypertensive encephalopathy
4. Pre-eclampsia and eclampsia
5. Acute pressure response to an exogenous agent

We will explain each of the items in more detail in this essay.

### 1. Pheochromocytoma (benign or malignant)

Pheochromocytoma is a catecholamine-producing tumor originated from chromaffin cells that are located in the adrenal medulla and sometimes the extra-adrenal tissues.^2^ It is known as “The tumor of tens”; 10% are extra-adrenal, 10% are bilateral, 10% are malignant, 10% are found in asymptomatic patients, and 10% are hereditary.^3^ Some of these percentages have changed according to more recent studies.

The incidence of pheochromocytoma is 0.5% in patients with hypertensive symptoms. The traditional triad of the disease are episodic headache, sweating, and palpitations.^4^ Other characteristic symptoms include visual disturbances, abdominal and chest pain, anxiety, nausea, and vomiting. The evaluation may reveal bilateral papilledema, orthostatic hypotension, and transient EKG changes. Patients usually have a persistent hypertension; although, detection of normal or low blood pressure in pheochromocytoma is not particularly rare.^5^ For example, patients who have a genetic predisposition to pheochromocytoma tend to be normotensive. Moreover, the tumor is probably smaller in size in this group of patients. These include about 24% of all the patients (contrary to the previously mentioned 10%).^6^

The most well-known familial syndromes associated with pheochromocytoma are Von Hipple-Lindau (VHL), multiple endocrine neoplasia type 2 (MEN2), and Neurofibromatosis type 1(NF1); all of which have an autosomal dominant mode of inheritance.^2^

If one suspects the disorder in a patient on the basis of clinical presentations, the next diagnostic step is biochemical testing. The classic test is a measurement of urinary catecholamines and their metabolites, which have a sensitivity and specificity of 98%.^7^ The alternative is to measure plasma metanephrines that is extremely sensitive, and is suggested by some authorities as screening test. It has a sensitivity of about 99%, but the specificity is lower (85%–89%).^8^ In fact, the detection of excessive catecholamines or their metabolites in the urine or serum is the basis of diagnosis. Imaging studies should be used to localize the tumor.^9^ CT and MRI are useful in localizing the majority of tumors, except the smaller ones that are usually found in the hereditary form of disease.^10^ MIBG and PET scan are complementary imaging tools. These techniques are sensitive in bilateral tumors or in situations when the patient is suspicious of malignancy. Furthermore, they are useful when the tumor is greatly suspected in clinical settings, but the inspections have been nonconclusive.^6^

Medical therapy has a crucial role in symptomatic treatment, but surgery is essential for the cure.

Prevention of intraoperative catastrophic hypertensive crisis and the hypotension immediately after the surgery must be considered.^11^

As mentioned earlier, headache is an important symptom of pheochromocytoma. 51-80% of patients with pheochromocytoma describe headaches which are often paroxysmal. Paroxysmal headaches are severe, may have a persistent or pulsatile quality, and are usually located in the frontal or occipital region. Headache is typically accompanied by perspiration, palpitations, anxiety, and facial pallor, and is known to occur simultaneously with sudden elevation of blood pressure.^12^ In about 70% of patients the duration of headaches is less than1 hour.

Diagnostic criteria for headache in pheochromocytoma are included in Table 1.^1^

**Table 1:** Diagnostic criteria for headache in pheochromocytoma

A. Intermittent discrete attacks of headache accompanied by at least one of the following and fulfilling criteria C and D.
1. Sweating
2. Palpitations
3. Anxiety
4. Pallor
B. Pheochromocytoma demonstrated by biochemical investigations, imaging and/or surgery
C. Headache develops concomitantly with abrupt rise in blood pressure
D. Headache resolves or markedly improves within 1 hour of normalization of blood pressure

### 2. HTN crisis without encephalopathy

Hypertensive crisis is an acute and severe elevation in blood pressure often described as SBP > 180 mmHg, or DBP > 120 mmHg. However, according to the International Headache Classification (IHCD-2), the definition is somewhat different (SBP > 160 mmHg, or DBP > 120 mmHg). It is discussed in the two subtypes of hypertensive urgency and hypertensive emergency depending on absence or presence of end organ damage, respectively.^13^ In addition, higher levels of blood pressure are observed in emergent condition. It is estimated that one to two percent of the hypertensive patients experience at least one episode of crisis in their life.^14^ The majority of patients have a history of established primary or secondary HTN.^15^

There is a peak of incidence for HTN crisis between the ages of 40 and 50 years.^16^ It is more common in males and African Americans. Patients with uncontrolled HTN may be at a higher risk of developing a crisis, although data is inadequate in this regard.^17^

Some disorders and medical conditions are related to hypertensive crisis; renal disorders (vascular or parenchymal), Cushing's disease, poor medication compliance, cocaine and PCP abuse, abrupt withdrawal of some antihypertensive agents such as clonidine, and etcetera.^18^

The precise mechanism of hypertensive crisis is not well understood. It is presumed that a diffuse vascular constriction caused by humoral factors is the trigger for the hypertensive process. The consequence is vascular endothelial injury and activation of coagulation factors leading to fibrinoid necrosis of the arterioles. Renin-angiotensin system and most vasoconstrictors, such as norepinephrine and vasopressin, add to the process and eventually we have rapid blood pressure elevation and end organ ischemic damage.^19^

Headache, epistaxis, faintness, psychomotor agitation, chest pain, dyspnea, neurologic deficits, and less often arrhythmias and paresthesias are the clinical presentations.^20^ Intracranial hemorrhage, acute aortic dissection, acute MI, and acute kidney injury are rare but serious complications of hypertensive crisis.^21^

Hypertensive crisis needs to be promptly treated in order to prevent lethal complications.

Hypertensive urgencies, once no serious end organ damage suspected, may be managed as outpatients with oral antihypertensive agents. However, hypertensive emergencies necessitate admission to the intensive care unit and aggressive therapy. Short-acting titratable intravenous antihypertensives, including labetalol, esmolol, fenoldopam, nicardipine, sodium nitroprusside, and etcetera, are the drugs of choice. Sodium nitroprusside has the risk of toxicity and should be used cautiously.^18^

Headache, as a symptom, has a prevalence of about 20% in hypertensive urgency. It has a bilateral throbbing quality. Helpful diagnostic criteria have been validated for the headaches based on the hypertensive crisis without encephalopathy regardless of the etiology.

Diagnostic criteria for headaches in HTN crisis without encephalopathy are listed in Table 2.^1^

**Table 2:** Diagnostic criteria for headaches in HTN crisis without encephalopathy

A. Headache with at least one of the following characteristics and fulfilling criteria C and D:
1. Bilateral
2. Pulsating quality
3. Precipitated by physical activity
B. Hypertensive crisis defined as a paroxysmal rise in systolic (to > 160 mmHg) and/or diastolic (to > 120 mmHg) blood pressure but no clinical features of hypertensive encephalopathy
C. Headache develops during hypertensive crisis
D. Headache resolves within 1 hour after normalization of blood pressure
E. Appropriate investigations have ruled out vasopressor toxins or medications as causative factors

### 3. Hypertensive encephalopathy

Accelerated HTN, also named hypertensive encephalopathy, may result in disturbance of consciousness. It is usually related to hypertensive emergency, as already mentioned. In fact the encephalopathy syndrome (headache, neurologic deficit, seizure, and confusional state) is a type of organ damage in the setting of hypertensive emergency.^22^

Clinical presentation of hypertensive encephalopathy is due to the effect of high blood pressure on the blood brain barrier that impairs its integrity and leads to cerebral hyperperfusion and finally brain edema occurs. In a normal situation, the well-known cerebral autoregulatory effect inhibits the blood brain barrier from damage in a certain range of systolic blood pressure increments. Indeed, the autoregulatory mechanism is active as long as the mean arterial pressure (MAP) is kept in a range of 50-150 mmHg. In chronic hypertensive patients, as a consequence of adaptation, this range is shifted to higher levels of MAP.^23^

In extremely rapid accelerations of blood pressure, MAP exceeds the defined level and we have an orthostatic leakage of plasma across the capillaries. The condition is transient and predominant in posterior cerebral regions and is usually called posterior reversible encephalopathy syndrome (PRES).^24^

The etiology of hypertensive encephalopathy is almost the same as those for hypertensive crisis without encephalopathy but in a more severe manner. Clinical signs and symptoms of hypertensive encephalopathy and probable accompanying signs and symptoms are listed in Table 3.^25^ It is mandatory to look for serious complications such as cerebral, cardiovascular, ophthalmic, and renal events.

**Table 3:** Clinical manifestations of end organ damage from hypertensive emergency

Central nervous system	dizziness, nausea and vomiting, confusion, weakness, encephalopathy, ICH, SAH, ischemic stroke
Eyes	ocular hemorrhage, exudates, papilledema, blurred vision, loss of sight
Heart	angina, ACS, LVF, PE, aortic dissection, cardiogenic shock
Kidneys	hematuria, proteinuria, pyelonephritis, elevated serum Cr and BUN, ARF

ACS: Acute Coronary Syndrome, ARF: Acute Renal Failure, BUN: Blood Urea Nitrogen, Cr: Creatinine, ICH: Intracranial Hemorrhage, LVF: Left Ventricular Failure, PE: Pulmonary Edema, SAH: Subarachnoid Hemorrhage

Apparently, headache can be an important guiding symptom of the disorder and, has a vascular origin. Criteria for headache in hypertensive encephalopathy are listed in Table 4.^1^

**Table 4:** Criteria for headache in hypertensive encephalopathy

A. Headache with at least one of the following characteristics and fulfilling criteria C and D:
1. Diffuse pain
2. Pulsating quality
3. Aggravated by physical activity
B. Persistent blood pressure elevation to > 160/100 mmHg with at least two of the following:
1. Confusion
2. Reduced level of consciousness
3. Visual disturbances (other than those of typical migraine aura) including blindness
4. Seizures
C. Headache develops in close temporal relation to blood pressure elevation
D. Headache resolves within 3 months after effective treatment and control of hypertension
E. Other causes of the neurological symptoms have been excluded

### 4. Pre-eclampsia and eclampsia

Pre-eclampsia affects about 5-8% of pregnancies.^26^ It is part of a spectrum known as the hypertensive disorders of pregnancy.^27^ Pre-eclampsia is characterized by hypertension, abnormal peripheral edema, and proteinuria.

The patient may complain of new persistent epigastric pain and vomiting. In examination, brisk tendon reflexes, and swelling of hands, face, or feet can be detected. A rise in serum creatinine, thrombocytopenia, and liver enzymes are the associated lab data abnormalities.^28^

A combination of maternal and placental factors contributes to the condition. Lack of previous pregnancy (nulliparity), obesity, a history of pre-eclampsia, and maternal medical comorbidities (diabetes mellitus, hypertension, antiphospholipid antibody syndrome, connective tissue disorders, or other thrombophilic situations) have been posed as the etiology. Patient's age is also a determinant factor; pre-eclampsia is more common before 18 and after 40 years of age.^29^ The incidence of pre-eclampsia is 9% in obese women in comparison with the 2% risk of incidence among the normal population.^30^

Pre-eclamptic women are subjected to cardiovascular complications. Studies show an approximately 4-fold increase in the risk of subsequent development of hypertension later in life, and a 2-fold increase in the risk of ischemic heart disease, venous thrombosis, and stroke in pre-eclamptic women.^31^

Magnesium sulfate is the drug of choice for women with pre-eclampsia since it halves the risk of eclampsia.^28^

Neurologic signs and symptoms are common presentations of the disorder, including severe persistent headache, visual defects (such as blurred vision, diplopia, or floating spots), confusion, depression of consciousness, and finally may lead to seizures or eclampsia. In this regard the manifestations are much the same as hypertensive encephalopathy.^32^ Pre-eclamptic headaches can be diagnosed using the criteria listed in Table 5.^1^

**Table 5:** Criteria for headaches in pre-eclampsia

A. Headache with at least one of the following characteristics and fulfilling criteria C and D:
1. Bilateral
2. Pulsating quality
3. Aggravated by physical activity
B. Pregnancy or puerperium (up to 4 weeks postpartum), and pre-eclampsia defined by both of the following:
1. Hypertension (> 140/90 mmHg) documented on two blood pressure readings at least 4 hours apart
2. Urinary protein excretion > 0.3 g per 24 hours
C. Headache develops during periods of high blood pressure
D. Headache resolves within 7 days after effective treatment of hypertension
E. Appropriate investigations have ruled out vasopressor toxins, medications, or pheochromocytoma as causative factors

Whenever pre-eclampsia is superimposed by seizure or coma, or both, then it is named eclampsia.^26^ The underlying pathophysiology, to some extent, seems to be similar to hypertensive encephalopathy, which is cerebral overperfusion due to failure of autoregulatory response.^33^ In fact, these two circumstances are part of a spectrum known as posterior reversible encephalopathy syndrome (PRES) which has characteristic features. Vasogenic brain edema is the underlying cause of this syndrome and accounts for the loss of consciousness.^32^

Imaging study, especially brain MRI, shows edema in the cortex and subcortical white matter that predominantly involves the occipital lobes.^24^ No reliable serum markers have been identified to predict the risk of eclampsia in pregnant women.^34^

It is very important to manage pre-eclampsia appropriately, as soon as the diagnosis is established, in order to prevent its progression to eclampsia, because the latter is harmful for both mother and fetus. There is absolute evidence that early administration of magnesium sulfate lessens the occurrence of eclampsia by more than 50% in pre-eclamptic women. Furthermore, it has been proved that magnesium sulfate is a more efficient drug to control eclamptic seizures in comparison with many antiepileptic drugs.^35^ Labor inductions are essentially the definite treatment for eclampsia.

Headache is a common warning symptom of eclampsia that should be inquired in any pregnant woman visited in perinatal care sessions. Criteria for headache in eclampsia are listed in Table 6.^1^

**Table 6:** Criteria for headache in eclampsia

A. Headache with at least one of the following characteristics and fulfilling criteria C and D:
1. Bilateral
2. Pulsating quality
3. Aggravated by physical activity
B. Pregnancy or puerperium (up to 4 weeks post-partum), and eclampsia defined by all of the following:
1. Hypertension (> 140/90 mmHg) documented on two blood pressure readings at least 4 hours apart
2. Urinary protein excretion > 0.3 g per 24 hours
3. A seizure has occurred
C. Headache develops during periods of high blood pressure
D. Headache resolves within 7 days after effective treatment of hypertension
E. Appropriate investigations have ruled out vasopressor toxins, medications, or phaeochromocytoma as causative factors
F. Stroke has been excluded

### 5. Acute pressure response to an exogenous agent

In any patient who complains of headache and we detect a high blood pressure, it is essential to review his/her drugs and ask the patient about cocaine abuse. Acute pressure response to an exogenous agent may cause a hypertensive emergency. The most famous of the agents include cocaine, amphetamines, oral contraceptives, and monoamine oxidase inhibitors (MAOI), especially when interacting with tyramine-containing foods. Hypertensive emergency may also occur following the withdrawal of beta-blockers, alpha-stimulants (e.g. clonidine), or alcohol.^36^ Criteria related to this type of headache are listed in Table 7.^1^

**Table 7:** Criteria for headache due to acute pressure response to an exogenous agent

A. Headache, no typical characteristics known, fulfilling criteria C and D
B. An appropriate agent or toxin has been administered or ingested and an acute rise in blood pressure has occurred
C. Headache develops in close temporal relation to the acute rise in blood pressure
D. Headache resolves within 24 hours after normalization of blood pressure
E. No other mechanism for the headache is apparent
